# 2112. Incidence and outcomes of infections in liver transplant recipients during the first year post-transplant.

**DOI:** 10.1093/ofid/ofac492.1733

**Published:** 2022-12-15

**Authors:** Rodolfo M Alpizar-Rivas, Rohith Palli, Michael Croix, Paritosh Prasad, Purba Gupta, Sally Chuang

**Affiliations:** University of Rochester, Rochester, New York; University of Rochester, Rochester, New York; University of Rochester, Rochester, New York; University of Rochester, Rochester, New York; University of Rochester, Rochester, New York; Universtity of Rochester, Rochester, New York

## Abstract

**Background:**

Despite the use of antimicrobial prophylaxis in liver transplant recipients (LTR), infection remains the most likely cause of death (18.94%) in the first year. There is limited data describing the characteristics, & infections of LTR while hospitalized in the first year post-transplantation. We aimed to review the incidence and outcomes of infections in LTR in the first year post-transplant during hospitalization or re-hospitalization.

**Graph 1.**
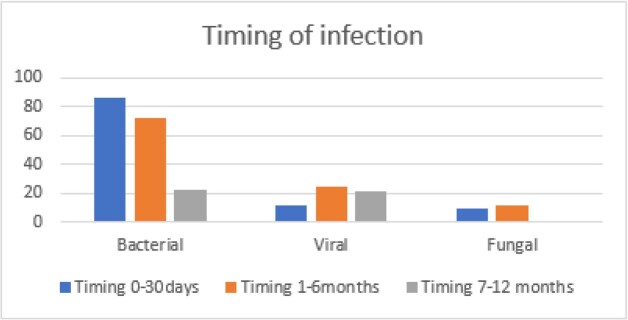
Y-axis; Number of cultures collected, X-axis; type of organism dived over time.

Site of bacterial infection

**Table 1.**
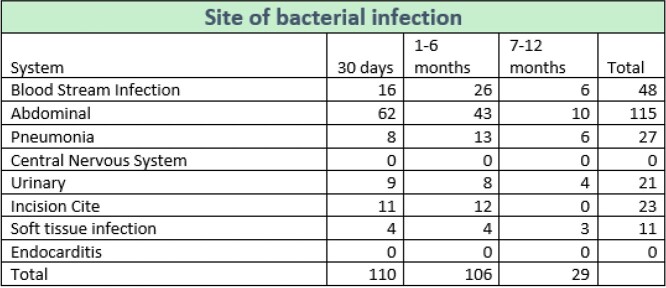
Number of bacterial infections divided by site/system, involved compared to timing of infection.

**Methods:**

We performed a single-center retrospective cohort study at The University of Rochester. Electronic medical records of 298 first time adult LTR between 03/5/2011 and 04/01/2020 were reviewed. Demographic and comorbidity data was obtained at time of transplant. Microbiologic data and outcomes were obtained during the first year post-transplant. We compared mortality between those with infection and those without using log-rank survival analysis.

**Graph 2.**
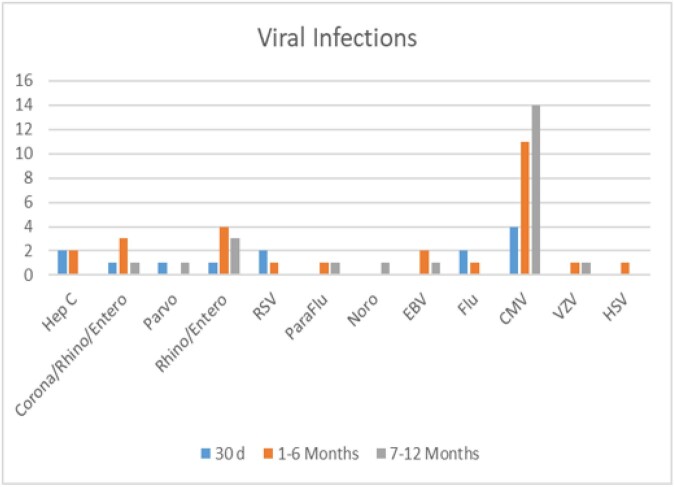
Y-Axis; Number of patients with diagnosed viral infection. X-axis virus divided over time.

**Results:**

160 (53.7%) patients suffered at least 1 infection while hospitalized in the first year post-transplant. There were 178 bacterial infections in 130 patients of which 115 were intra-abdominal (table 1). The most common organism was *Enterococcus sp.* (n=102), 65 were vancomycin resistant (VRE). The most common site of fungal infection was abdominal (n=13) and the most common organism was *C. glabrata* (n=9). The most common viral infection was CMV at 7-12 months although other viral infections occurred between 1-6 months (graph 2).

In a univariate chi-square model, bacterial infection at any time was associated with all-cause mortality in the first year (CI 1.7-8.9 p-adj= 0.002). Fungal infection between 2-6 months was also associated with increased mortality (CI 2.7-12.2 p-adj= 0.0005).

**Conclusion:**

Bacterial infections are the most common type of infection in LTR and occur more frequently during the first 6 months. Bacterial infection at any point was independently associated with increased mortality in the first year post-transplant. The most common organism was *Enterococcus* with a high rate of VRE which should be taken into consideration for empiric coverage at our institution. Fungal infection between 2 to 6 months was associated with increased mortality. We observed that early bacterial and fungal infections are markers of poor prognosis.

**Disclosures:**

**All Authors**: No reported disclosures.

